# The Microbiome of the Middle Meatus in Healthy Adults 

**DOI:** 10.1371/journal.pone.0085507

**Published:** 2013-12-30

**Authors:** Vijay R. Ramakrishnan, Leah M. Feazel, Sarah A. Gitomer, Diana Ir, Charles E. Robertson, Daniel N. Frank

**Affiliations:** 1 Department of Otolaryngology-Head and Neck Surgery, University of Colorado, Aurora, Colorado, United States of America; 2 Division of Infectious Diseases, University of Colorado, Aurora, Colorado, United States of America; 3 Microbiome Research Consortium, University of Colorado, Aurora, Colorado, United States of America; 4 Department of Molecular, Cellular, and Developmental Biology, University of Colorado, Boulder, Colorado, United States of America; Hospital of the University of Pennsylvania, United States of America

## Abstract

Rhinitis and rhinosinusitis are multifactorial disease processes in which bacteria may play a role either in infection or stimulation of the inflammatory process. Rhinosinusitis has been historically studied with culture-based techniques, which have implicated several common pathogens in disease states. More recently, the NIH Human Microbiome Project has examined the microbiome at a number of accessible body sites, and demonstrated differences among healthy and diseased patients. Recent DNA-based sinus studies have suggested that healthy sinuses are not sterile, as was previously believed, but the normal sinonasal microbiome has yet to be thoroughly examined. Middle meatus swab specimens were collected from 28 consecutive patients presenting with no signs or symptoms of rhinosinusitis. Bacterial colonization was assessed in these specimens using quantitative PCR and 16S rRNA pyrosequencing. All subjects were positive for bacterial colonization of the middle meatus. *Staphylococcus aureus, Staphylococcus epidermidis* and *Propionibacterium acnes* were the most prevalent and abundant microorganisms detected. Rich and diverse bacterial assemblages are present in the sinonasal cavity in the normal state, including opportunistic pathogens typically found in the nasopharynx. This work helps establish a baseline for understanding how the sinonasal microbiome may impact diseases of the upper airways.

## Introduction

Rhinitis and rhinosinusitis, whether acute or chronic, are highly prevalent disease processes. Chronic rhinosinusitis alone affects 14% of the population and accounts for $8.6 billion in direct expenditures annually in the United States [1]. Chronic rhinosinusitis (CRS) symptom severity can be as severe as quality of life alterations found in major diseases like congestive heart failure, angina, chronic obstructive pulmonary disease, and back pain [2,3]. Many medical and surgical therapies have been utilized with varying degrees of success, however the etiology and susceptibility for such diseases are still poorly understood. Bacterial superinfection of damaged mucosa has been described as the most important cause of acute rhinosinusitis, but its role in CRS pathogenesis is less clear [4]. Although acute rhinitis and rhinosinusitis is most commonly infectious, CRS is known as a multifactorial and idiosyncratic disease process where bacterial infection or colonization may play some role in the initiation or sustenance of the inflammatory response.

Although the anterior nasal cavity and nasopharynx are known bacterial reservoirs, it has long been held that the sinuses were “sterile” in the healthy state. Most of our understanding on bacteria in rhinitis and rhinosinusitis comes from culture-based studies, which may not be a sensitive enough method to accurately identify microbial presence [5]. However, the paradigm of presence or absence of a single pathogen as the source of disease appears to be changing. The “microbiome” concept that the bacterial community composition contributes to health and disease states has recently been suggested for the upper and lower airway [6-8]. The microbiome as a community of functional organisms within the host has great genetic potential to serve as a disease modifier. Recent data from gastrointestinal and allergy research groups have demonstrated that not only is the microbiome relevant for pathogen exclusion, it is essential in shaping the host immune system through pathways of relevance in CRS (dendritic cells, Th17, Treg cells) [9-13]. Although much attention has been devoted to the role of commensal gut microbiota in shaping early immunologic development and susceptibility to inflammatory and allergic diseases [14], our upper aerodigestive tract is similarly highly exposed to the environment and is likely rapidly colonized with commensals early in life. 

Many types of infections result from initial mucosal invasion by a pathogen, and the chronic inflammation that results from complex interaction between the mucosal barrier, innate and adaptive immune systems, and the inflammatory response [15] is nowhere more noticeable than the sinonasal cavity. In fact, several preliminary studies have demonstrated a link between upper and lower airway bacterial composition and bronchial hyperreponsiveness, peripheral eosinophilia, and total IgE [16,17]. Study in germ-free mice has demonstrated a key role for commensal microbial colonization in the regulation of Th2 allergic inflammation of the airways [18]. With the introduction of modern molecular techniques and data analysis capabilities, several studies have detected a much more diverse population of bacteria in the sinuses of CRS patients. In one study of control and CRS patients, culture identified bacteria in 81% of patients and provided a mean of 1.4 organisms, whereas bacterial DNA sequencing identified bacteria in all patients with a mean of 10 organisms identified [19].

Although there is considerable pressure to examine disease states, it is logical to begin with disciplined examination of the normal state in order to define the effects of microbiota on disease and pathogenesis, and in fact this is the recommendation of the NIH Human Microbiome Project Working Group [6, http://commonfund.nih.gov/hmp/]. The aim of this study was to assess the middle meatus microbiome of healthy control patients to understand the baseline degree of richness and diversity, and assess for similarities or differences between patients.

## Materials and Methods

### Study design and Population

This cross-sectional study was approved by the Institutional Review Board of the University of Colorado (COMIRB protocol number 11-1442), and written informed consent was obtained from all patients. Healthy patients without rhinosinusitis who underwent either (1) endoscopic sinus surgery for approach to a small unilateral skull base lesion, (2) endoscopic orbital surgery, or (2) endoscopic septoplasty for nasal airway obstruction, were enrolled and subjected to sampling of the disease-free middle meatus. All patients with skull base or orbital lesions had radiographically normal preoperative CT scans on the side that was sampled (opposite to tumor), and no endoscopic evidence of inflammatory disease outside of the known lesion. Control patients who underwent septoplasty for nasal airway obstruction had otherwise normal preoperative CT scans. Patients less than 18 years of age, with recent antibiotic use or use of intranasal medications (within one month of surgery), with a history of prior sinonasal surgery, those with a known immunodeficiency, cystic fibrosis or autoimmune disease were excluded from the study. Patients diagnosed with allergic rhinitis, CRS, and/or asthma according to established criteria were also excluded [20-22].

### Sample Collection

Samples were collected at the onset of surgery at University of Colorado Hospital between January 2011 and June 2012. CultureSwabs^TM^ (BD, Franklin Lakes, NJ) for DNA extraction were endoscopically guided deeply within the middle meatus, rotated at least 5 full turns until visibly saturated, and placed on ice upon collection and frozen at -80°C until DNA extraction.

### DNA extraction

A phenol:chloroform bead-beating method was used to extract total genomic DNA from the swab heads, as previously described [23]. All DNA extraction and PCR steps were performed in a HEPA-filtered laminar flow hood decontaminated by ultraviolet light. DNAs were precipitated by the addition of 0.5 volume ammonium acetate (7.5 M) and 1 volume of 100% isopropanol, incubated at -80° C for 10 minutes, and centrifuged (>14,000 × *g*; 25 min). Nucleic acid pellets were washed with 250 μl of 70% ethanol and centrifugation (>14,000 × *g*; 5 min), lyophilized to dry, then re-suspended in 30μl of sterile 1x Tris-EDTA (pH 8.0), and stored at –80°C until PCR processing. 

### Quantitative PCR

A duplex quantitative PCR (QPCR) assay using previously published oligonucleotide primers for Total Bacteria (16S rRNA gene, FAM reporter) [24] was conducted on an ABI 7300 thermocycler. PCR reactions contained 10μl DyNAmo ColorFlash Probe qPCR Mastermix with ROX (Finnzymes Oy, Espoo, Finland), 3μl water, and 1μl each 20x PrimeTime^TM^ primer/probe set (Integrated DNA Technologies, Inc., Coralville, Iowa), and 5μl of DNA template. Thermocycling was conducted as follows: 7 min initial denaturation at 95°C, followed by 40 cycles of 15 s at 95°C and 1 min at 60 °C. Standard curves were generated and absolute quantitation of DNA copy numbers per volume of template were obtained. 

### 454 pyrosequencing

Amplicons of the V1V3 variable region of the bacterial 16S rRNA gene (~500bp; primers 27FYM+3 and 515R) were generated via broad-range PCR (30-36 cycles) using 5’-barcoded reverse primers [25-27]. Pooled amplicons were provided to the Center for Applied Genomics at the University of Toronto for pyrosequencing on a 454/Roche Life Sciences GS-GLX instrument using titanium chemistry (Roche Life Sciences, Indianapolis, IN). All pyrosequences were screened for nucleotide quality: bases at 5’ and 3’ ends with mean Q<20 over a 10 nucleotide window, sequences with less than 200 nucleotides, and sequences with more than one ambiguous nucleotide were discarded [27,28]. Mean trimmed sequence length was ~340 bp. Genus-level taxonomic calls were produced by the ribosomal database project (RDP) classifier, which performs naïve Bayesian taxonomic classification versus a training set [29]. Species level taxonomy precision was obtained via BLAST (Basic Local Alignment Search Tool, http://blast.ncbi.nlm.nih.gov/Blast.cgi) [30] against a database of sequences obtained from Silva version 104 [31] tagged as isolates and reported results demanded at least 99% sequence identity over 95% of sequence length. Standard biological indices of diversity, richness, and evenness were conducted with the BIODIV software tool (www.phyloware.com) embedded within the sequence analysis pipeline [32]. These indices were estimated through bootstrap resampling (1000 replicates) and rarefaction of the OTU distributions obtained from each specimen. The Good’s index of each sequence library was >95%, indicating that most of the biodiversity was captured in each library. All DNA sequence data were deposited in the NCBI short read archive (Project PRJNA221204).

### Statistics

Fisher exact tests were performed for categorical outcomes (presence/absence of condition, OTU prevalence). Comparison of percent abundances of particular species between subjects was performed using a Wilcoxon Rank Sum Test of the relative abundance values. Associations between continuous variables were tested by linear regression. Differences in the compositions of microbiomes (i.e. operational taxonomic unit [OTU] distributions taken as a whole) between patient groups were measured by the Bray-Curtis index using the adonis function of the R package vegan, which performs a nonparametric multiple analysis of variance with adjustment for covariates [33,34]. A label permutation test with 10,000 replicates was used to assign a p-value to the difference in OTU counts between groups of samples. All tests of null hypotheses were evaluated at α = 0.05. All statistical analyses were performed using Explicet (www.explicet.org), the R statistical package (v.2.14.0, Institute for Statistics and Mathematics, Wien, Austria), or Microsoft Excel.   

## Results

**Figure 1:**
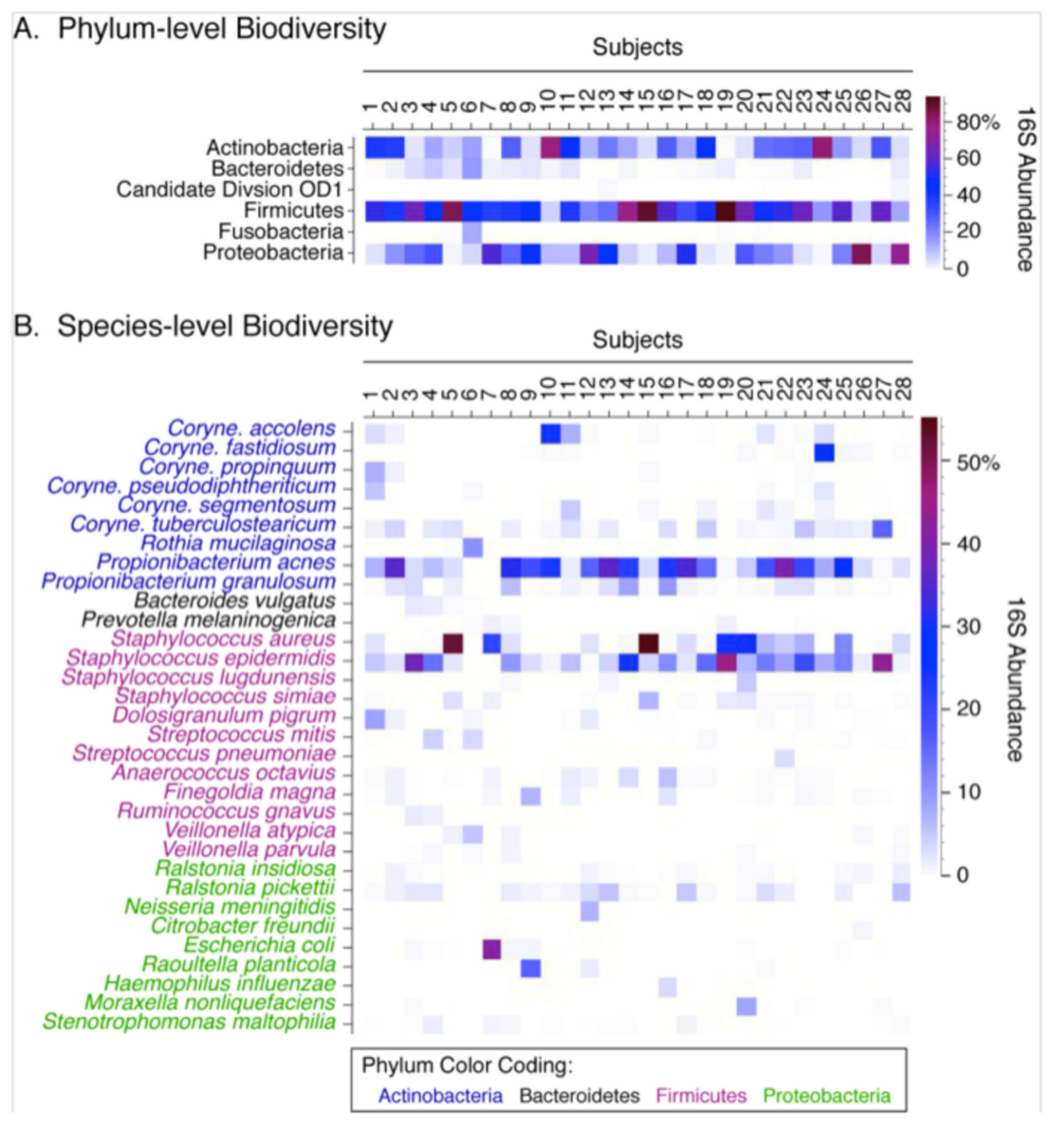
Phylum- and species-level diversity. (A) Phylum-level classification for each subject demonstrates community diversity, but also variability between subjects. Only phyla with median relative abundances greater than 0.5% are shown. (B) Species-level analysis with a minimum 0.5% abundance demonstrates diversity and variability between subjects.

Twenty-eight adult patients (18 male, 10 female; mean age 46.6 yrs, range 18-66 yrs) undergoing sinonasal surgery between January 2011 and July 2012 were included in the study. Of these, 19 (68%) underwent septoplasty for nasal obstruction and 9 (32%) underwent an endoscopic approach for management of skull base/orbital lesion. The initial assessment of overall bacterial loads in middle meatus specimens was performed through quantitative PCR using pan-bacterial primers [24], which demonstrated the presence of bacteria in all specimens. To determine the kinds and relative quantities of bacterial groups in the specimens, we performed broad-range PCR and pyrosequencing of bacterial 16S rRNA gene sequences. We generated a median of 1312.5 pyrosequences per sample (interquartile range 855.8 to 2230.8). Good’s coverage index, a measure of how completely each sample was sequenced, had a median value of 96.1% (interquartile range 95.1-97.0%, measured at the genus level). Operational taxonomic unit (OTU)-based analysis indicated a rich and diverse suite of bacteria (Table 1 and Figure 1A) dominated by the phyla Firmicutes, Proteobacteria, and Actinobacteria, each of which was detected in all subjects. The phylum Bacteroidetes also was present in the majority of subjects (83%), although at much lower relative abundance (2.5% of rRNA sequences). 

**Table 1:** Abundant Taxa within the Middle Meatus of Healthy Subjects (N = 28).

**Taxonomy^*1*^**		**Prevalence^*2*^**	**Relative Abundance^*3*^**	**Respiratory Pathogen^*4*^**
Firmicutes		100.0%	48.1%	
	*Staphylococcus epidermidis*	*96.4%*	*11.0%*	
	*Staphylococcus aureus*	*67.9%*	*8.3%*	**Y**
	*Staphylococcus simiae*	*50.0%*	*0.7%*	
	*Anaerococcus octavius*	*64.3%*	*0.7%*	
	*Finegoldia magna*	*46.4%*	*0.6%*	
	*Dolosigranulum pigrum*	*46.4%*	*0.5%*	
	*Streptococcus mitis*	*46.4%*	*0.4%*	**Y**
	*Veillonella atypica*	*21.4%*	*0.3%*	
	*Staphylococcus lugdunensis*	*14.3%*	*0.3%*	
	*Steptococcus sanguinis*	*39.3%*	*0.1%*	**Y**
	*Streptococcus oralis*	*25.0%*	*0.1%*	**Y**
	*Enterococcus faecalis*	*10.7%*	*0.1%*	**P**
**Proteobacteria**		**100.0%**	**25.2%**	
	*Escherichia coli*	*35.7%*	*1.6%*	**P**
	*Ralstonia pickettii*	*78.6%*	*1.5%*	
	*Raoultella planticola*	*10.7%*	*0.7%*	
	*Ralstonia insidiosa*	*60.7%*	*0.4%*	
	*Moraxella nonliquefaciens*	*17.9%*	*0.3%*	
	*Neisseria meningitidis*	*3.6%*	*0.3%*	**Y**
	*Stenotrophomonas maltophilia*	*60.7%*	*0.2%*	**P**
	*Haemophilus influenzae*	*7.1%*	*0.1%*	**Y**
	*Enterobacter aerogenes*	*10.7%*	*0.1%*	**P**
	*Moraxella catarrhalis*	*14.3%*	*0.03%*	**Y**
**Actinobacteria**		**100.0%**	**23.2%**	
	*Propionibacterium acnes*	*92.9%*	*14.7%*	
	*Corynebacterium tuberculostearicum*	*71.4%*	*2.0%*	
	*Propionibacterium granulosum*	*82.1%*	*1.7%*	
	*Corynebacterium accolens*	*53.6%*	*1.6%*	
	*Corynebacterium fastidiosum*	*32.1%*	*1.1%*	
	*Rothia mucilaginosa*	*28.6%*	*0.4%*	
	*Corynebacterium segmentosum*	*32.1%*	*0.4%*	
	*Corynebacterium propinquum*	*21.4%*	*0.4%*	
	*Corynebacterium pseudodiphtheriticum*	*39.3%*	*0.4%*	
**Bacteroidetes**		**83.3%**	**2.5%**	
	*Bacteroides vulgatus*	*21.4%*	*0.2%*	**P**
	*Bacteroides spp.*	*21.4%*	*0.1%*	**P**
	*Prevotella spp.*	*35.7%*	*0.3%*	**P**
**Fusobacteria**		**42.9%**	**0.6%**	
	*Fusobacterium nucleatum*	*7.1%*	*0.02%*	**P**
**Candidate-division-OD1**		**21.4%**	**0.1%**	
**Cyanobacteria**		**14.3%**	**0.04%**	
**Tenericutes**		**7.1%**	**0.1%**	
**Candidate-division-TM7**		**7.1%**	**0.03%**	
**Chlamydiae**		**10.7%**	**0.02%**	
**Elusimicrobia**		**14.3%**	**0.03%**	

^1^ Phyla and top 35 most abundant species detected in the sinuses of 28 healthy adults by culture-independent 16S rRNA sequence analysis.

^2^ Prevalence of taxon in study population (percentage of positive subjects).

^3^ Mean relative abundance of taxa, normalized to total number of sequences in each subject.

^4^ Potential (P) and known (Y) respiratory pathogens present in >10% of subjects. Respiratory pathogens identified from references 4,35–37.

**Figure 2:**
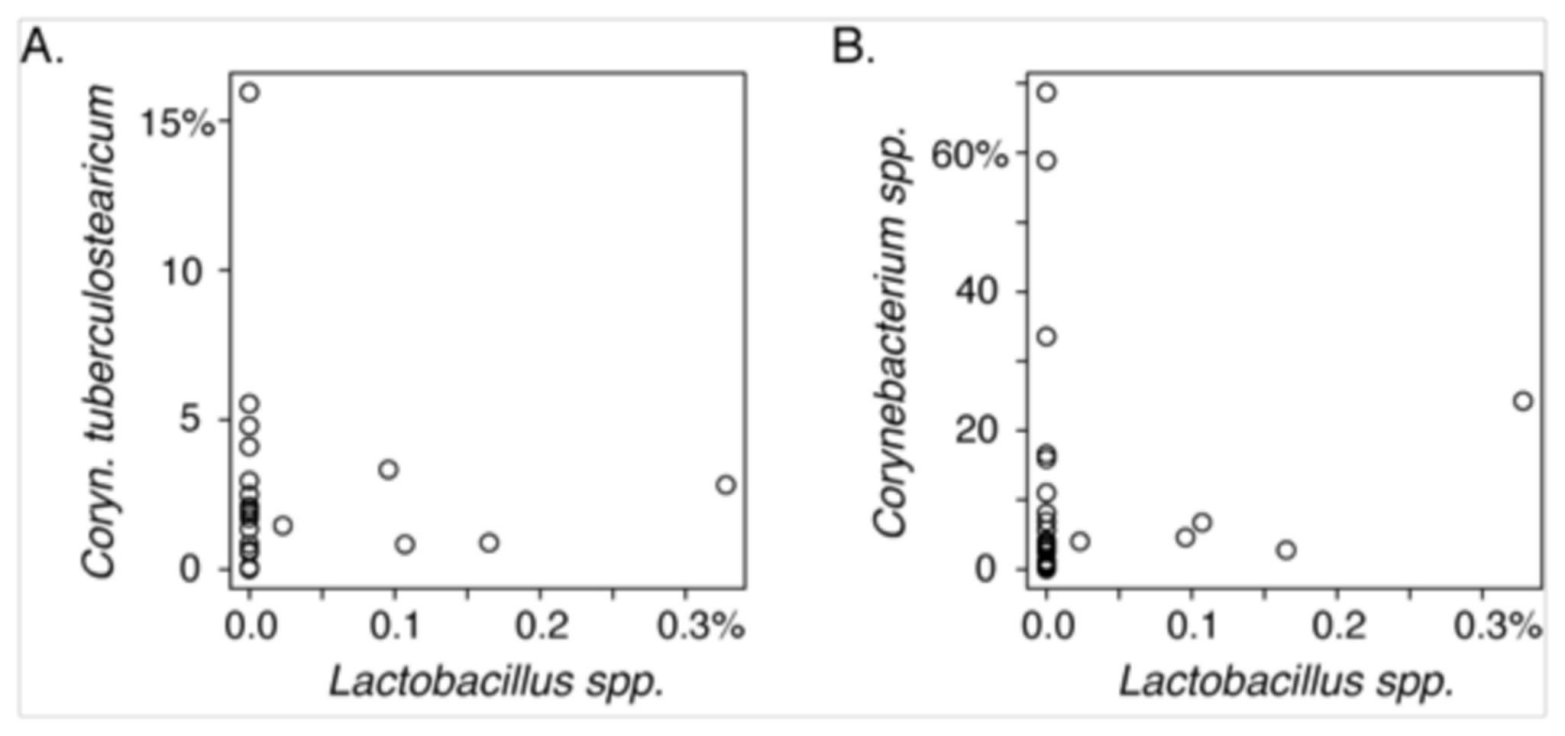
Correlation between relative abundances of Corynebacteria and Lactobacilli. 16S rRNA sequence abundances of *Lactobacillus* *spp*. and (A) *Corynebacterium tuberculostearicum* and (B) *Corynebacterium* *spp.* X- and Y-axes represent the percent abundances of the specified OTUs, normalized to total sequence counts. Each circle represents a study participant.

At the species level, high prevalences of *Staphylococcus epidermidis* (96.4%)*, Staphylococcus aureus* (67.9%), and *Propionibacterium acnes* (92.9%) were observed (Table 1, Figure 1B); these three species also exhibited the highest average relative abundances (11.0%, 8.3%, and 14.7% of rRNA sequences, respectively) among the species observed in the middle meatus. A variety of corynebacterial species also were observed, with a collective prevalence of 92.9 % (26/28 positive subjects) and moderate relative abundance (8.9% of rRNA sequences). *Corynebacterium tuberculostearicum* was the numerically dominant corynebacterial species observed in this healthy cohort, with a mean relative abundance of 2.0% of rRNA sequences (range 0.0% to 15.9%). Abreu et al recently reported a negative correlation between *C. tuberculostearicum* and lactobacilli in the sinuses that suggested a protective role for lactobacilli in non-CRS control subjects, compared with CRS patients [8]. In our population, however, lactobacilli were a minor component of the overall sinonasal microbiome of our healthy cohort, with relative abundances ranging from 0.0% to 0.3% of rRNA sequences (mean of 0.04%). Neither *C. tuberculostearicum* nor the genus *Corynebacterium* was correlated in abundance with the genus *Lactobacillus* (Figure 2). 

Overall, the prevalences and abundances of *S. epidermidis, P. acnes*, and Corynebacteria were similar to our previously reported analyses of the sinonasal microbiome in CRS [23], and the anterior nares microbiome in healthy individuals [28]. *S. aureus* was observed in approximately the same proportion of subjects in both CRS (66.7%) [23] and healthy subjects (67.9%; this study). Both of these values are in contrast to the lower frequency of detection of *S. aureus* in the anterior nares (16.7%) [28]. In addition to *S. aureus*, we detected a variety of confirmed or potential opportunistic pathogens of the airways including *Streptococcus pneumoniae, Neisseria meningiditis, Haemophilus influenzae*, and *Moraxella catarrhalis*, but all were at low relative abundance (Table 1) [4,35-37]. Several organisms were discovered in this normal population which are typically associated with disease—most notably *Stenotrophomonas maltophilia*, Streptococcus sp, Enterobacter sp, Fusobacterium sp, and several anaerobes in the Bacteroidetes phylum—suggesting that either these are not necessarily true pathogens, or that they may inhabit select individuals in low relative abundance and carry a potential for acute overgrowth in certain conditions. 

The population-level summary statistics described above belie substantial subject-to-subject heterogeneity in the relative abundances of even the most prevalent phyla and species (Figures 1A and 1B). Thus, several subjects (e.g., subjects 3, 5, 14, 15, and 19) were dominated by Firmicutes, mainly members of the genus Staphylococcus, while for other subjects the Actinobacteria (e.g. subjects 10 and 24) or Proteobacteria (e.g., subjects 26 and 28) were most abundant (Figure 1A). Because of the cross-sectional study design, it is unclear whether this heterogeneity represents (1) temporal instability (unlike the stable communities observed in the anterior nares) [28], (2) specific consortia that are uniquely adapted to different subjects, analogous to enterotypes described within the human gut microbiome [38], or (3) the potential for direct niche competition between species. 

**Figure 3:**
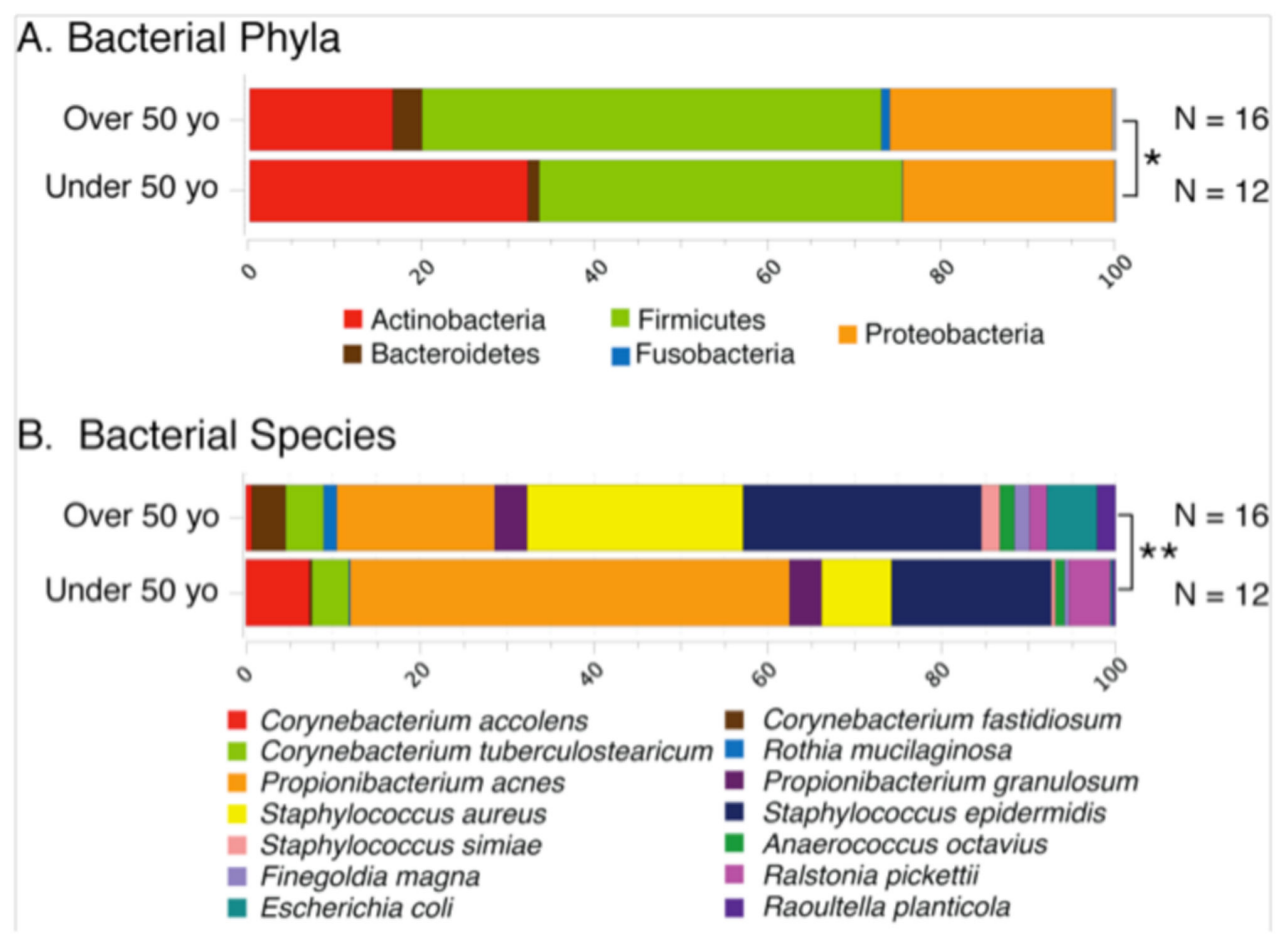
Age-associated differences in the healthy microbiome. Differences in the phylum-level (panel A) and species-level (panel B) percent relative abundances of 16S rRNA sequences between subjects categorized by age (over or under 50 years of age) are shown. Only taxa with percent relative abundances greater than 0.5% are included; the abundant species are normalized to 100% in order to better depict between-group differences. Multivariate analyses of microbiome datasets revealed significant differences at both the phylum-level (*: p = 0.03) and species-level (**: p = 0.004).

To understand the source(s) of variation in the healthy middle meatus microbiome, we performed exploratory analyses of a variety of demographic and clinical factors that might affect microbiota composition (Tables 2 and 3). At the species level, both age (categorized by decade) and residence (coded by zip code) were significantly associated with the microbiome, whereas age was also significantly associated with the phylum-level microbiome (Tables 2 and 3, Figure 3). Older individuals harbored diminished levels of Actinobacteria (Figures 3A), including members of the genera Corynebacterium and Propionibacterium (Figure 3B), and concomitantly greater abundances of Fusobacteria (Figure 3A) and *S. aureus* (Figure 3B). Although not statistically significant, older subjects were characterized by a much greater range of biodiversity indices. Together, these observations suggest that age may lead to disruption of the middle meatus microbiome, perhaps as a result of immune senescence.

**Table 2:** Univariate Analysis of Microbiome Composition in Healthy Subjects.

	***P* Values**		
**Variable**	**Species**	**Phylum**	**Notes**
Over 50 y	***0.004***	***0.03***	Over or under 50 years old
Allergies	0.58	0.66	
Asthma	0.96	0.83	
Decade	***0.03***	***0.06***	Age in decades
Diabetes	0.34	0.77	
Ethnicity	0.85	0.68	
Gender	0.42	0.32	
Residence	***0.05***	0.24	Encoded by zipcode
Saline washes	0.86	0.74	
Smoking (NVR, CUR, FRM)	0.18	0.51	Never, current, former smoker
Smoking (NVR, EVR)	0.15	0.41	Never or ever smoker
Steroid spray	0.50	0.45	

**Table 3:** Multivariate Analysis of Microbiome Composition in Healthy Subjects.

	***P* Values**		
**Variable**	**Species**	**Phylum**	**Notes**	
Over 50 y	***0.003***	***0.04***	Over or under 50 years old	
Residence	***0.098***	0.22	Encoded by zipcode	
Smoking (NVR, EVR)	0.34	0.68	Never or ever smoker	

**Figure 4:**
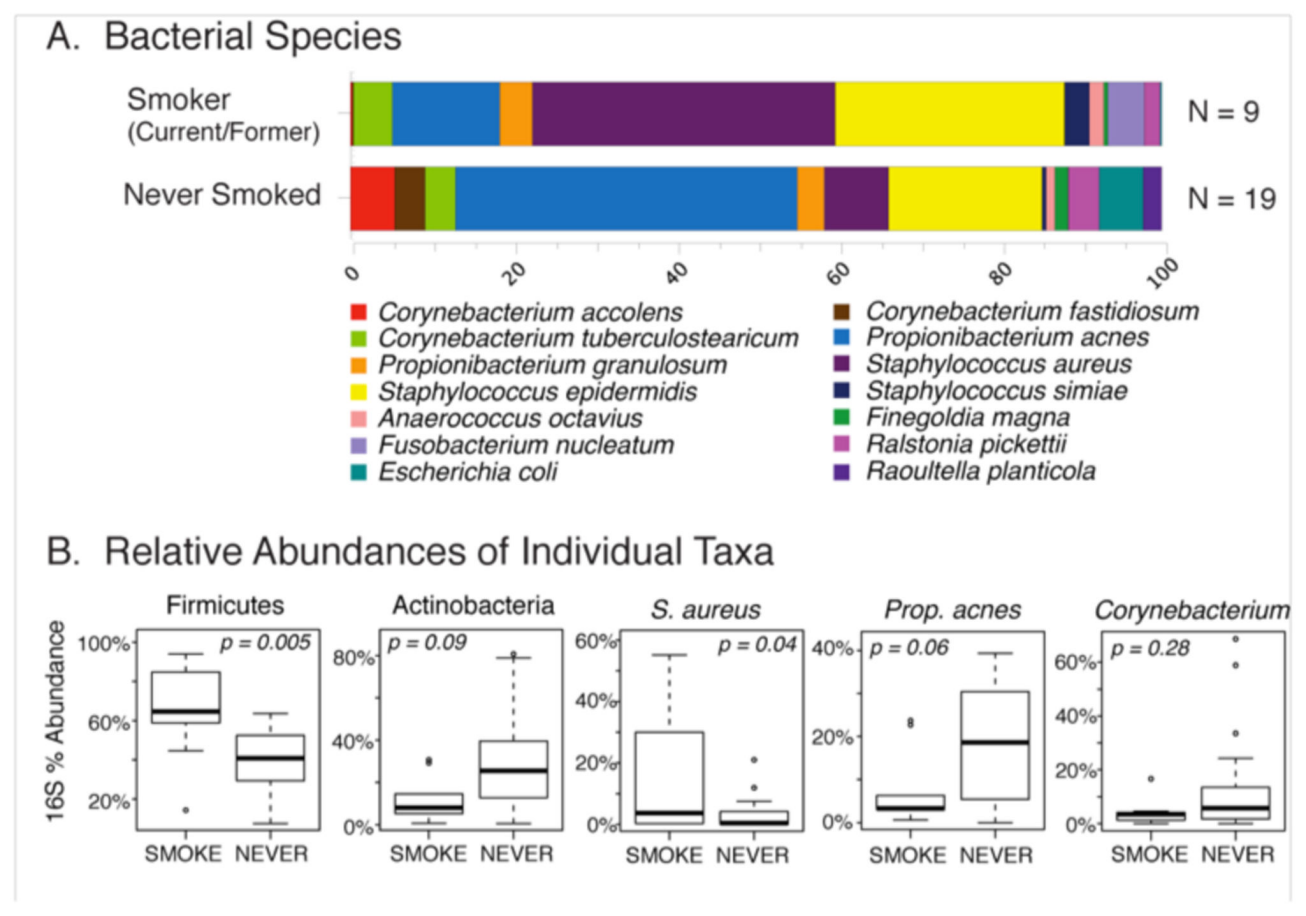
Effects of smoking on the healthy microbiome. Differences in the species-level (panel A) percent relative abundances of 16S rRNA sequences between subjects categorized by history of smoking are shown. Only taxa with percent relative abundances greater than 0.5% are included; the abundant species are normalized to 100% in order to better depict between-group differences. Although multivariate analyses of microbiome datasets did not reveal a significant association between smoking and microbiome composition (p = 0.15), select taxa differed significantly in percent relative abundance between smoking categories (panel B).

Although smoking was not a significant determinant of overall microbiome composition (Table 2), the distribution of species in the microbiomes of smokers (current or former) differed qualitatively from that of non-smokers (Figure 4). Consequently, we examined the predominant individual OTUs for differences in relative abundance between smokers and non-smokers. Among smokers there was a significant increase in the phylum Firmicutes (p = 0.005), especially the species *S. aureus* (p = 0.04), compared to non-smokers. In contrast, members of the phylum Actinobacteria including *P. acnes* and Corynebacteria trended toward decreased levels in smokers.

## Discussion

Our findings indicate that a rich and diverse community of bacteria inhabits the middle meatus of healthy patients. Although some taxa (*P. acnes*, *S.* epidermidis, *S.* aureus) dominated the microbiomes of multiple individuals, notable variability was observed between subjects (Figure 1), implying that future upper airway and sinus microbiome study will require large patient cohorts. Interestingly, several known and potential pathogens were recovered at varying abundances. Perhaps most notable is the presence of *S. aureus* in 68% of samples at 8% abundance, whereas the remaining pathogens were typically found at lower relative abundance. These findings align with the recent publication by the Human Microbiome Project Consortium [39], which reported that multiple bodily subsites in healthy subjects are characterized by signature taxa, but that the relative representation of taxa between individuals was variable. The HMP study also noted that known pathogens were rarely encountered, but that many opportunistic pathogens were broadly distributed at low relative abundances. The middle meatus, anatomically, is located at the junction of the nasal cavity and anterior sinuses within a region referred to as the osteomeatal complex—a region which has long been considered relevant in the pathogenesis and severity of rhinosinusitis [54-57]. Our data, taken in this context, would suggest that species considered pathogenic in acute or chronic rhinosinusitis may be present in low abundances in healthy patients and have the potential to create disease after an acute alteration in the stable baseline microbiome (*dysbiosis*). In addition, many of these species that are uncommonly found on clinical culture—such as *S. maltophilia* and many anaerobes—are often present in health and may not truly be pathogenic.

Clearly, differences are noted between normal patients’ microbiota. A certain degree of variability has been noted in the microbiome of healthy patients in large studies of other bodily subsites, although certain taxa typically dominate a given anatomical site [39]. Several studies have suggested that microbiomes in healthy states are relatively stable over time, although this has been debated [47,48]. It may be that certain people have a more “mobile” microbial fingerprint even in healthy states, and there may be particular times in which susceptibility to infection rises, for instance following disruption of the normal composition of the microbiome. Interestingly, preliminary studies of the GI response to antibiotic challenge (“resilience”) have shown great variation in the degree of response and time to restoration of the initial baseline microbiome following treatment [49,50]. Initial microbiome richness may be a factor that (1) limits the potential for infection by a particular pathogen and (2) resists mobility of the core microbiota, thus offering the hypothesis that people with a rich and dense baseline microbiome are potentially less susceptible to infection [51]. 

Some limitations of the current study must be acknowledged. Although the presumed “normal” patients included in this study did not have symptomatic or objective evidence of mucosal inflammation within the sinonasal cavity, it is conceivable that some mild associated inflammation was present, as we did not rule this out with any quantitative molecular techniques. Another potential limitation of this study could be the introduction of nasal microorganisms into specimens during endoscopic sinus surgery. However, our sampling method attempted to minimize contamination from the anterior nasal cavity by careful endoscopic direction and placement of the swab, a technique that is used clinically to obtain a representative sample of the underlying paranasal sinuses [40]. Comparison to previous studies of the normal anterior nares [28,39] showed clearly different microbiota in the current study, supporting the notion that our method of sampling the middle meatus was able to avoid contamination from the anterior nasal cavity. Furthermore, these findings suggest that sinus infections may result from pathogens often not found in the anterior nasal cavity. Finally, the microbes present in the middle meatus may not be entirely representative of the underlying sinuses. When compared to the “gold standard” of maxillary sinus puncture and aspirate, prior culture-based studies of middle meatus sampling in acute rhinosinusitis have demonstrated an accuracy in the 80-90% range [41-45], leading to the clinical practice recommendation for culture-directed antibiotics in the specialty care of CRS [46]. However, in the era of more sensitive culture-independent microbiologic techniques [5], and with the understanding that subtle alterations in the microenvironment may result in alterations in the local microbial communities, this acceptance must be critically re-examined. 

Several limitations of human microbiome investigation in general are also present in the current study. Although stability of a niche microbiome over time in the healthy state has been suggested [39], there are likely at least small changes that occur around a homeostatic “fingerprint” for a given body subsite in a given person. This, in addition to interpersonal variation even in the healthy state, underscores the need for recruitment of large cohorts with multiple sampling points over time in future studies. Furthermore, the current study does not address the potential role of rare community members, or complex interplay between bacteria, viruses, and fungi. A number of key microbiome functions may be performed by rare community members [39], and consequently it has been suggested that we may be “essentially blind” to many of the functions of our microbial ecosystems [52]. A recent study utilizing a phylogenetic microarray (16S rRNA PhyloChip, Affymetrix Corporation, Santa Clara, California, USA) for investigation of the rarer constituents of the sinonasal microbiome found decreased richness, evenness, and diversity in CRS patients when compared to controls [8]. This technique utilized a high-density microarray corresponding to 8,500 bacterial taxa, which may be useful for detection of less abundant bacteria within the community. The authors found an increased relative abundance of *C. tuberculostearicum* and a decrease in *L. sakei* in chronic rhinosinusitis patients, a pattern which our study of the normal middle meatus does not seem to initially support. This discrepancy could be explained by differences in methodology (sequencing vs microarray, sampling site and technique), differences in patient population (age, race, climate), or that six of the ten chronic rhinosinusitis patients in the study of Abreu et al. [8] were treated with antibiotics preoperatively. 

Although there are a number of challenges in the study of the paranasal sinus microbiome, there is *significant* room for innovation. Alterations in the microbiome in the sinonasal cavity may suggest a role in upper airway disease susceptibility and pathogenesis, the initiation and sustenance of inflammation, or alternatively may result from disease or treatments administered in disease states. Initial investigation relies upon examination of the normal state, as there is a clear role for commensals in pathogen exclusion and in the modulation of inflammation. Future studies require longitudinal sampling to determine microbiome stability and resilience to perturbation such as viral or bacterial infection, as well as the administration of topical and systemic therapies such as rinses, corticosteroids, and antibiotics. 

## Conclusions

Examination of middle meatus and sinuses in both healthy and diseased patients has demonstrated that the paranasal sinuses are not sterile. Rather, a complex bacterial milieu exists within the human upper airway that may be altered in a number of conditions. These alterations, or *dysbioses* [53], have the potential to influence many inflammatory pathways currently implicated in chronic rhinosinusitis, such as those involving innate immunity, immune cell recruitment, wound healing and extracellular matrix remodeling, and epithelial cell integrity. Bacterial community dynamics may link to other local and systemic inflammatory pathways, and further work is required to examine these possibilities. 
